# Salivary flow rate, pH, and concentrations of calcium, phosphate, and sIgA in Brazilian pregnant and non-pregnant women

**DOI:** 10.1186/1746-160X-2-44

**Published:** 2006-11-28

**Authors:** Maria I Rockenbach, Sandra A Marinho, Elaine B Veeck, Laura Lindemann, Rosemary S Shinkai

**Affiliations:** 1 Department of Oral Surgery, Pontifical Catholic University of Rio Grande do Sul, Porto Alegre, Brazil; 2 Graduate Program in Oral Medicine, Pontifical Catholic University of Rio Grande do Sul, Porto Alegre, Brazil; 3 Private practice, Porto Alegre, Brazil; 4 Department of Prosthodontics, Pontifical Catholic University of Rio Grande do Sul, Porto Alegre, Brazil

## Abstract

**Background:**

Studies on salivary variables and pregnancy in Latin America are scarce. This study aimed to compare salivary flow rate, pH, and concentrations of calcium, phosphate, and sIgA of unstimulated whole saliva in pregnant and non-pregnant Brazilians.

**Methods:**

Cross-sectional study. Sample was composed by 22 pregnant and 22 non-pregnant women attending the Obstetrics and Gynecology Clinics, São Lucas Hospital, in Porto Alegre city, South region of Brazil. Unstimulated whole saliva was collected to determine salivary flow rate, pH, and biochemical composition. Data were analyzed by Student t test and ANCOVA (two-tailed α = 0.05).

**Results:**

No difference was found for salivary flow rates and concentrations of total calcium and phosphate between pregnant and non-pregnant women (p > 0.05). Pregnant women had lower pH (6.7) than non-pregnant women (7.5) (p < 0.001), but higher sIgA level (118.9 mg/L) than the latter (90.1 mg/L) (p = 0.026).

**Conclusion:**

Some of the tested variables of unstimulated whole saliva were different between pregnant and non-pregnant Brazilians in this sample. Overall, the values of the tested salivary parameters were within the range of international references of normality.

## Background

Hormonal changes in females may affect the physiology of the entire body including the oral cavity. Besides the direct effect on the metabolism of periodontal tissues, pregnancy, menstruation, and hormone replacement therapy may induce short-term changes in salivary flow rates, buffering capacity, and biochemical composition [1-5]. Changes in salivary composition and flow rates may compromise the integrity of the soft and hard tissues in the oral cavity, because saliva functions include food and bacteria clearance, mastication and digestion, lubrication, antimicrobial defense, and buffering effect [6,7]. Saliva is composed of water and organic and inorganic molecules, but a large intra- and inter-subject variability in composition is reported [2,6].

Most studies focusing on the influence of pregnancy and hormonal alterations on salivary characteristics were performed in European countries, and some reference standards for normality [6,8] are derived from data obtained in specific populations. The Latin American literature on this topic is scarce. A preliminary search of the electronic database Latin American and Caribbean Literature on the Health Sciences (LILACS) using the terms "saliva" and "pregnancy" resulted in eleven articles published in the last 20 years, and only one evaluated salivary flow rates and/or pH in pregnant women [9]. A similar search of PubMed MEDLINE yielded another study in Latin American women [10]. Therefore, it is difficult to compare results from populations with potential differences of genetics and dietary habits.

This cross-sectional study aimed to compare the salivary flow rates, pH, and concentrations of calcium, phosphate, and secretory immunoglobulin A (sIgA) of the unstimulated whole saliva in pregnant and non-pregnant women, residents in the metropolitan area of Porto Alegre city, in the South region of Brazil. The a priori hypothesis was that there are differences of salivary flow rate, pH, and biochemical composition of saliva between pregnant and non-pregnant women.

## Methods

A convenience sample was composed of 44 women aged between 18 and 38 years-old, selected among the patients attending the Obstetrics and Gynecology Clinics, São Lucas Hospital, in Porto Alegre city, South region of Brazil. All of the subjects were healthy, functioning individuals attending the clinics for regular exam, with no complaint of xerostomia. Twenty-two consecutive pregnant women (mean age: 27.9 years-old), between the fifth and ninth month of pregnancy, comprised the pregnant group. Exclusion criteria were high-risk pregnancy and unwillingness to participate in the study. The comparison group was composed of 22 non-pregnant women (mean age: 29.5 years-old), who attended the same clinics for routine exam. The research protocol was in compliance with the Helsinki Declaration and was approved by the institutional review board of the Pontifical Catholic University of Rio Grande do Sul. All subjects signed an informed consent form before the study procedures.

A structured questionnaire was used to collect data on oral hygiene habits (frequency of tooth brushing, use of dental floss), professional counseling on oral health and hygiene, and presence of gingival bleeding. Data on medical conditions and use of medications were retrieved from the subject's medical charts.

### Sialometrical analysis

The collection of unstimulated whole saliva was performed under resting conditions between 07:30 and 10:30 am, at least 1 h after eating, following standard procedures [11]. Subjects were asked to sit passively and expectorate into pre-weighed plastic containers for 5 min as the saliva accumulated in the floor of the mouth. Salivary samples were collected on ice, and the volume was determined gravimetrically: The weight of each saliva sample (g) was equated to volume (mL), since the specific gravity of saliva is 1.0 [12,13]. Salivary flow rates were expressed as mL/min.

### Sialochemical analysis

Saliva samples were centrifuged (centrifugal force: 1,000 g) to remove bacteria and other extraneous material. The resulting clarified fluid was used for the biochemical assays to measure salivary pH and the concentrations of the following parameters: total calcium, inorganic phosphate, and sIgA.

Salivary pH was determined by means of micro-electrodes of a blood gas analyzer that measured the hydrogen ion concentration. Salivary pH values were reported as the log of the mean hydrogen ion activity.

Total calcium was determined using the CPC photometric method based on the work of Gitelman [14]. Calcium ions form a violet complex with *o-*cresolphthalein complexone in an alkaline medium. The reaction is measured colorimetrically with CPC at 545 nm. The intensity of the colour is directly proportional to calcium concentration in the sample.

Inorganic phosphate was determined using the phosphomolybdate/UV method of Daly and Ertingshausen [15], which relies on the formation of a UV absorbing complex between phosphorus and molybdate. Inorganic phosphorus reacts with ammonium molybdate in the presence of sulfuric acid to form an unreduced phosphomolybdate complex which is measured as an endpoint reaction at 340 nm.

Secretory IgA was measured by a radial-immunodiffusion method [16] using specific antibodies to form precipitation rings in agarose gels. The diameter of the ring formed is quantitatively related to the concentration of the salivary parameter analyzed.

### Statistical analysis

Data were analyzed by descriptive statistics, and comparisons between the pregnant and non-pregnant groups were performed using Student t test for independent samples for salivary flow rate and analysis of covariance (ANCOVA) for salivary pH, and concentrations of calcium, phosphate, and sIgA. For ANCOVA, the fixed factor was pregnancy status (pregnant vs. non-pregnant) and the covariate was salivary flow rate. All statistical tests were two-tailed, and a P-value of 0.05 was considered statistically significant for rejection of the null hypothesis.

## Results

Pregnant women had lower salivary pH (6.7) than non-pregnant women (7.5) (p < 0.001), but higher sIgA level (118.9 mg/L) than the latter (90.1 mg/L) (p = 0.026). Salivary flow rates and levels of total calcium and phosphate were not statistically different between pregnant and non-pregnant women (p > 0.05) (Table 1). All values were within the international references of normality (Table 2).

**Table 1:** Salivary flow rate, pH, and concentrations of calcium, phosphate, and sIgA in pregnant and non-pregnant women.

***Variable***	***Pregnant (n = 22)***		***Non-pregnant (n = 22)***		P*
	***Mean***	***SD***	***Mean***	***SD***	

Salivary flow rate (mL/min)	0.59	0.37	0.64	0.33	0.652
PH	6.7	0.4	7.5	0.4	<0.001
Calcium (mmol/L)	1.1	0.3	0.9	0.4	0.252
Phosphate (mmol/L)	5.6	1.9	5.6	2.3	0.803
sIgA (mg/L)	118.9	47.0	90.1	35.0	0.026

* Student t test for independent samples (for salivary flow rate) and ANCOVA (for salivary pH, and concentrations of calcium, phosphate, and sIgA; covariate: salivary flow rate), two-tailed α = 0.05.

**Table 2:** Contrast of the values of the tested salivary variables with reference standards of normality.

***Variable***	***Minimum***	***Maximum***	***Mean***	***95% Confidence interval***	***Normal reference values***
***Pregnant (n = 22)***					
Flow rate (mL/min)	0.12	1.73	0.59	[0.43–0.75]	0.1 – 0.5*
PH	5.9	7.6	6.7	[6.5–6.9]	5.5 – 7.4**
Calcium (mmol/L)	0.5	1.8	1.1	[0.9–1.2]	0.5 – 2.7 *
Phosphate (mmol/L)	2.4	9.1	5.6	[4.8–6.5]	1.9 – 22.9 *
sIgA (mg/L)	12.0	185.0	118.9	[98.0–139.7]	60 – 300 ***
					
***Non-Pregnant (n = 22)***					
Flow rate (mL/min)	0.20	1.53	0.64	[0.50–0.78]	0.1 – 0.5 *
PH	6.6	8.0	7.5	[7.3–7.6]	5.5 – 7.4**
Calcium (mmol/L)	0.4	1.8	0.9	[0.8–1.1]	0.5 – 2.7 *
Phosphate (mmol/L)	2.6	12.7	5.6	[4.6–6.7]	1.9 – 22.9 *
sIgA (mg/L)	34.0	173.0	90.1	[74.6–105.6]	60 – 300 ***

*Edgar (1992); **Thylstrup & Fejerkov (1994); ***Dade Behring^®^

In relation to oral hygiene habits, 16 pregnant (73%) and 15 non-pregnant (68%) patients used to brush their teeth at least three times a day. Most women (16 pregnant [73%] and 17 non-pregnant [77%]) had received professional orientation of oral hygiene care and followed these recommendations, but some did not use dental floss (9 pregnant [41%] and 4 non-pregnant [18%] women). The pregnant patients who did not use dental floss had gingival bleeding, and two of them reported that the bleeding started during the gestational period.

Regarding intake of medication, 11 pregnant and 15 non-pregnant women did not use any medication. Some pregnant women under medication reported use of ferrous sulphate and analgesics. Non-pregnant women used mainly oral contraceptives; analgesics also were recorded.

## Discussion

In this sample salivary pH and concentration of sIgA were different between pregnant and non-pregnant Brazilians, but no significant difference was found for salivary flow rates of unstimulated whole saliva or concentrations of calcium and phosphate. We chose to collect unstimulated whole saliva because this type of saliva predominates during most part of the day and is important for maintenance of oral health, reflecting a physiological status of the oral cavity and of the entire body [17]. However, collection of true unstimulated saliva is difficult due to interferences of environmental stimuli, which may determine a broad range of salivary flow rates. For this reason, some researchers prefer to use stimulated saliva. In an update review on saliva for the FDI, it was stressed that the limits of the normal reference range of flow rates of saliva are still controversial [17]. Although we followed standard procedures for collection of saliva samples [11], we found a broad range of salivary flow rates for unstimulated whole saliva, which could potentially affect our results. For example, Rudney [2] reported that the concentration of slgA is negatively correlated with flow rate in unstimulated whole saliva and in either weakly or strongly stimulated parotid saliva. Furthermore, the influence of salivary flow rate on biochemical composition may be different according to the pregnancy status as Kivela et al. [18] found that the concentration of salivary carbonic anhydrase VI had a weak positive correlation with flow rate in late pregnancy but not in postpartum. Because there was a numerical trend of higher salivary flow rate in the non-pregnant group, we compared the pregnant and non-pregnant groups adjusting for salivary flow rate by using analysis of covariance for salivary pH, and concentrations of calcium, phosphate, and sIgA. Therefore, we removed any possible variation attributable to flow rate from the other variables [2].

In our study, as salivary flow rates did not differ with pregnancy status, we checked the medications taken by the study subjects to rule out any possible drug-related reduction of salivary flow rates [19]. Most non-pregnant women were taking oral contraceptives, but secretion rate of saliva does not seem to be affected by intake of this type of drug [20] or by estrogen status [21]. Previous studies with unstimulated and stimulated whole saliva also did not show any significant modifications of flow rates during pregnancy or in the post-partum period [22,23]. Conversely, some studies did find lower salivary flow rates in pregnant women compared with non-pregnant women [9,24], but all values of salivary flow rates were within the accepted normal range.

Although no difference of salivary flow rates was observed between the two groups, salivary pH was lower in pregnant (6.7) than in non-pregnant (7.5) women. In the latter, salivary pH was marginally above the upper limit of normality (7.4) according to international references. These values are similar to those reported by González et al. [9] for stimulated saliva in pregnant (pH 6.6) and non-pregnant Mexicans (pH 7.1). Laine and Pienihäkkinen [22] also found that the salivary buffer effect increased significantly from late pregnancy to postpartum, but this change was not related to salivary flow rates, since both unstimulated and paraffin-stimulated flow rates remained unchanged.

We did not find any difference in the concentrations of calcium and phosphate between the pregnant and non-pregnant groups despite the difference in salivary pH. Laine [3] also stated that pregnancy per se does not induce significant withdrawal of calcium or other minerals from the teeth. On the other hand, Salvolini et al. [1] reported a significant decrease of calcium levels between the 21st and the 40th week of gestation in relation to controls, and a decrease of phosphate at the 21st week. Other studies have reported a direct relation between increase of pH and increase of concentration of sodium and bicarbonate in saliva [25,26]. However, the possible regulatory mechanism of electrolyte and mineral composition of saliva remains unclear.

In relation to sIgA, we found higher levels of sIgA in pregnant women, but the difference between the two groups (pregnant: 119.8 mg/L versus non-pregnant; 90.1 mg/L) was minor. Secretion and synthesis of sIgA may be affected by stress, physical exercises, medications, menstrual cycle, and pregnancy [27]. The hormonal changes during pregnancy may have altered the sIgA levels because the production of estrogen and progesterone increases gradually until the eighth month of pregnancy, and both hormones modulate the immune system during the gestational period [3]. Widerström and Bratthall [24] also found increase of salivary concentration of sIgA during pregnancy, delivery and post-partum period, but not in non-pregnant women during the several phases of the menstrual cycle. As salivary flow rate of pregnant women was slightly lower than that of non-pregnant counterparts, the authors suggested that the increased level of sIgA was not influenced by salivary flow rate. This relation was also observed in our results. In our sample, because of the negligence of some pregnant patients with oral hygiene, the concentration of sIgA may also have increased due to an increase of the number of oral microorganisms as sIgA is the main adaptive immune mechanism in the oral cavity [27].

The cross-sectional design of this study does not allow inference of causal relationships, but our data corroborate some results of previous studies conducted in other parts of the world. Overall, the values of salivary variables analyzed in this sample of pregnant and non-pregnant Brazilians did not differ from values reported for other populations and were within the international references of normality. Nevertheless, it should be noted that the interval of these reference values is considerably large, reflecting the large variation that is considered to be within the normal range. Therefore, it seems to be difficult to use some of these salivary parameters, e.g., salivary concentrations of sIgA or phosphate, as outcome measures or biomarkers to diagnose systemic disease and monitor general health [28] unless the condition under investigation leads to large alterations of concentration. The differences between pregnant and non-pregnant women detected in our study and in previous papers are small in absolute values and would not impact clinical management of oral health in these groups. However, this situation may change when evaluating the effect of high-risk pregnancy in medically-compromised women on some salivary parameters.

## Conclusion

In summary, pregnant women had lower pH and higher sIgA concentration than non-pregnant women in this sample, but no significant difference was found for secretion rate of saliva or concentrations of calcium and phosphate. The values of the tested salivary parameters were within the international references of normality.

## Competing interests

The author(s) declare that they have no competing interests.

## Authors' contributions

MIR and SAM performed the data collection, data analysis, and wrote the manuscript. EBV conceived the study and its design. LL participated in the design of the study. RSS participated in the data analysis and preparation of the manuscript. All authors read and approved the final version of the manuscript.
